# Alternative splicing at GYNNGY 5′ splice sites: more noise, less regulation

**DOI:** 10.1093/nar/gku1253

**Published:** 2014-11-26

**Authors:** Meng Wang, Peiwei Zhang, Yang Shu, Fei Yuan, Yuchao Zhang, You Zhou, Min Jiang, Yufei Zhu, Landian Hu, Xiangyin Kong, Zhenguo Zhang

**Affiliations:** 1 State Key Laboratory of Medical Genomics, Institute of Health Sciences, Shanghai Institutes for Biological Sciences, Chinese Academy of Sciences and Ruijin Hospital, Shanghai Jiaotong University School of Medicine, Shanghai, People's Republic of China; 2 Graduate School of the Chinese Academy of Sciences, Beijing, People's Republic of China; 3 Institute of Molecular Evolutionary Genetics and Department of Biology, Pennsylvania State University, University Park, PA 16802, USA

## Abstract

Numerous eukaryotic genes are alternatively spliced. Recently, deep transcriptome sequencing has skyrocketed proportion of alternatively spliced genes; over 95% human multi-exon genes are alternatively spliced. One fundamental question is: are all these alternative splicing (AS) events functional? To look into this issue, we studied the most common form of alternative 5′ splice sites—GYNNGYs (Y = C/T), where both GYs can function as splice sites. Global analyses suggest that splicing noise (due to stochasticity of splicing process) can cause AS at GYNNGYs, evidenced by higher AS frequency in non-coding than in coding regions, in non-conserved than in conserved genes and in lowly expressed than in highly expressed genes. However, ∼20% AS GYNNGYs in humans and ∼3% in mice exhibit tissue-dependent regulation. Consistent with being functional, regulated GYNNGYs are more conserved than unregulated ones. And regulated GYNNGYs have distinctive sequence features which may confer regulation. Particularly, each regulated GYNNGY comprises two splice sites more resembling each other than unregulated GYNNGYs, and has more conserved downstream flanking intron. Intriguingly, most regulated GYNNGYs may tune gene expression through coupling with nonsense-mediated mRNA decay, rather than encode different proteins. In summary, AS at GYNNGY 5′ splice sites is primarily splicing noise, and secondarily a way of regulation.

## INTRODUCTION

Alternative splicing (AS) can produce more than one mRNA from the same gene (1–4), which greatly expands the complexity of transcriptome and proteome. Among various types of AS in mammals, exon-skipping is the most frequent form, followed by alternative 5′ and 3′ splice sites (5,6). A common type of alternative 5′ and 3′ splice sites uses two splice sites within a short distance (e.g. <6 nucleotides (7)), which are named tandem splice sites (8). For example, one frequent tandem 3′ splice site in eukaryotes is NAGNAG (9), where both AGs can be used as 3′ splice sites.

Recent advances in sequencing technologies have dramatically improved the capability of detecting novel splicing isoforms. For instance, over 95% of human multi-exon genes are reportedly alternatively spliced (5,10). Likewise, ≥42% multi-exon genes in *Arabidopsis thaliana* are subject to AS (11). These reports strongly suggest that AS is pervasive in eukaryote genomes.

A challenge, however, is to know the functions of these identified AS events. In fact, there has been a debate on whether AS at tandem splice sites are functional (referred to as regulation model) or splicing noise (referred to as noise model). Splicing noise occurs because splicing factors may bind nearby splice sites in a stochastic manner (12,13). The debate has focused on 3′ splice site NAGNAGs (14–17). Initially, the debate was on whether AS NAGNAGs were more conserved than constitutive ones and thus functional (14,17,18). Later, a study (7) using EST (expressed sequence tag) based splicing data and conservation among species claimed that a significant proportion of AS at tandem 3′ splice sites was likely splicing noise rather than providing regulation. Recently, a genome-wide study using RNA-seq data in humans and mice reported that ≤37% of AS events at NAGNAGs are strongly regulated across tissues and thus functional (16).

Despite previous great efforts, we do not have a comprehensive understanding of AS at tandem splice sites yet. First, only 3′ splice site NAGNAGs have been well studied. We have little knowledge of splicing at other types of splice sites, such as tandem 5′ splice sites. Encouragingly, studies have started to reveal features involved in regulation of 5′ splice sites (19), but these have been limited by small data size. Second, so far the argument for splicing noise at tandem splice sites is solely based on the mechanic model of splicing process and on lack of conservation for some sites; no direct evidence has been provided. Toward a better understanding of AS at tandem splice sites, we investigated 5′ splice site GYNNGYs (Y stands for C or T, and N stands for A, C, T or G) in humans and mice—the most common form of alternative 5′ splice sites (8,19). Our study, combined with previous studies, support that AS at tandem splice sites may be primarily accounted for by splicing noise, and secondarily by gene regulation.

## MATERIALS AND METHODS

### Data collection

We downloaded gene models from NCBI (20), Ensembl 71 (21) and UCSC (22) for humans, mice and rhesus monkeys (the genome versions for these species are hg19, mm10 and rheMac2).

We got the RNA-seq data for humans from the Illumina Body Map 2.0 project (NCBI GEO accession: GSE30611), which include 16 tissues (adipose, adrenal, brain, breast, colon, heart, kidney, liver, lung, lymph node, ovary, prostate, skeletal muscle, testes, thyroid and white blood cells). The sequencing reads are paired-ended but not strand-specific, each being 50 nt long. Each tissue has ∼80 million reads. We obtained the mouse and rhesus monkey RNA-seq data from a previous study (23), and we chose ‘mouse_a’ and ‘rhesus_b’ subsets for our analyses. For either species, the data include 9 tissues (brain, colon, heart, kidney, liver, lung, skeletal muscle, spleen and testes). The data are strand-specific and paired-ended. For mice, the read length and sequencing depth vary among tissues (heart and lung: 36 nts, ∼30 million reads per tissue; the other 7 tissues: 50 nts, 87–110 million reads per tissue). For rhesus monkey, all reads are 80-nt long, and each tissue has 100–110 million reads. The summary of the RNA-seq data is in Supplementary Table S1.

### Mapping RNA-seq reads to genomes

We used TopHat v2.0.8b (24) to map the raw reads in fastq format onto the corresponding reference genomes. The gene models from Ensembl 71, UCSC and NCBI Refseq were used to guide the mapping onto known transcripts. For rhesus monkey, we only used gene models from Ensembl 71 because of only a small number of annotated genes in NCBI and UCSC. We used the option ‘–no-coverage-search’ for TopHat to disable predicting new splice junctions based on ‘islands’ of mapped reads, but to allow getting new splice junctions through spliced mapped reads (see TopHat manual http://ccb.jhu.edu/software/tophat/manual.shtml).

To obtained gene expression levels, we used Cufflinks 2.1.1 (25) with the read alignments as input. The known transcripts annotated in Ensembl 71 were also given to the –GTF option as a guide for gene models.

### Identifying alternatively spliced GYNNGYs

First, we identified all splice sites based on the alignments between RNA-seq reads and the genomes. Briefly, introns were identified as insertions from the genome sequence when aligned to the reads, and splice junctions of these identified introns were in turn determined by looking at the intron's genomic locations. Then splice sites were extracted from the intronic sequences at the junctions. For non-strand-specific sequencing, before getting the splice sites, we had to determine the gene-coding strand from which the RNA-seq reads were transcribed. If the two nucleotides at the two ends of the identified ‘intron’ (genomic insertion in alignments) were ‘GT-AG’, ‘GC-AG’ or ‘AT-AC’, then the reads were from the strand where they were mapped, otherwise the reads were from the complementary strand of the mapped strand. Reads whose coding strands could not be determined were discarded. Second, to minimize the mapping errors, we required the reads supporting splice junctions (i) perfectly matched the genome (TopHat mapping quality score = 50), (ii) had at least six nucleotides on both joined exons and (iii) had no mismatches or indels in these six exonic nucleotides defined in (ii). For a GYNNGY 5′ splice site, if both GYs were supported as splice sites by mapped reads, the splice site was classified as AS GYNNGY, otherwise it was constitutive splicing GYNNGY. During the process, read mappings at a few splice junctions were ambiguous, because the nucleotide segment at the 3′-end of an exon matched the nucleotides at the 3′-end of its downstream intron or the segment at the 5′-end of an exon matched the 5′-end of the upstream intron. In both cases, mapping of the exonic segment could be uncertain. Therefore, this kind of splice junctions was excluded in our analyses.

**Figure 1.**
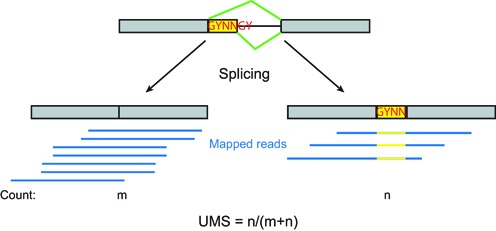
The diagram of AS at GYNNGY 5′ splice site as well as strategy of detecting such AS events by mapped reads. The formula for calculating the UMS is also given. The diagram shows the case of using the second GY as the minor form, but in reality the first GY can also be minor form.

For each AS GYNNGY, we measured the splice site usage as UMS, short for usage of minor splicing isoform. We identified the minor splice site of each AS GYNNGY by averaging the usages of either splice site across all tissues and regarding the one with the lower average usage as the minor form. This procedure consistently identified a minor form for each AS GYNNGY, and it is possible that the minor splice site becomes dominant in some tissues. After the minor splice site was determined, its UMS in each tissue was calculated as the number of reads supporting the splicing at the minor splice site divided by the total number of reads mapped to either splice site (Figure 1). The measurement equals the PSI (percent spliced-in) defined by a previous study (5) when the minor splice isoform is the one using the second GY, otherwise UMS = 1 − PSI. However, UMS is more informative in getting information of minor splice sites, which is needed in our analyses. ΔUMS for a GYNNGY is defined as the absolute difference between the maximum and minimum UMS across tissues.

For identifying the GYNNGYs whose splicing varies among tissues, we performed a permutated chi-squared test for each GYNNGY site. We included sites that had ≥10 mapped reads in ≥2 tissues, because 2 tissues is the minimum that the chi-squared test can be performed. We then shuffled the counts of reads across tissues and among splicing isoforms by requiring that the total numbers of reads assigned for each tissue and for each splicing isoform (summed over tissues) were the same as the original data. For each shuffled data, a *χ*^2^ value was calculated. We repeated this process 10^5^ times for each site, and counted the times *M* when the permutated *χ*^2^ values were greater than the *χ*^2^ value from the original data. A *P*-value was calculated as *M* divided by 10^5^. After that, the *P*-values were converted to Benjamini and Hochberg's false discovery rate (FDR) in *R* (26) with for multiple testing correction.

To infer an upper bound for the proportion of tissue-regulated AS GYNNGYs, we extrapolated the available data of 16 human tissues to an infinite number of tissues, by fitting the data to mathematical models. We tried to two models: Model 1, *y* ∼ *a* + *b* * exp(*c* * *x*), and Model 2, *y* ∼ *a* * *x* /(*b* + *x*), where *y* is the proportion of regulated sites and *x* is the number of used tissues, and *a*, *b*, *c* are parameters to be estimated in the fitting. Model 1 gave better fit than Model 2: the Akaike information criterion values are −299157 and −299082 for Model 1 and 2, respectively. The Model 1 also gave a higher correlation between observed and fitted values (Pearson's *R* = 0.9997131). Therefore, we used Model 1 to infer the upper bound of tissue-regulated AS GYNNGYs. We also tried the same approach on mouse data. However, we could not make reliable inference with only 9 tissues, because the proportion increases linearly with the number of tissues within this range (Supplementary Table S2). Our method does not imply that humans or mice have an infinite number of tissues, but uses a mathematical strategy to estimate an upper bound of regulated sites by extrapolating available data.

### Calculating 5′ splice site scores

We used the online tool MaxEntScan::score5ss (27) to estimate the splicing strength of 5′ splice sites (http://genes.mit.edu/burgelab/maxent/Xmaxentscan_scoreseq.html). Briefly, the 9-mer sequence at each splice site, including 3 nucleotides from the exon and 6 nucleotides from the downstream intron, was compared to a predefined probabilistic model representing the consensus of 5′ splice sites. The better the 9-mer matches the consensus, the higher the score is. We used all four models implemented in the tool (MAXENT, Maximum Entropy Model; MDD, Maximum Dependence Decomposition Model; MM, First-order Markov Model; and WMM, Weight Matrix Model), all giving similar results.

### Identifying motifs in the flanking regions of strongly regulated AS GYNNGYs

We firstly used MEME (version 4.10.0) (28) to identify motifs present in the downstream flanking 50-nt intronic regions of strongly regulated AS GYNNGYs. The parameters were ‘meme -dna -mod anr -minw 4 -maxw 10 -nmotifs 10 sequences.fa’, where the file ‘sequences.fa’ contained the intronic sequences in which the motifs were searched for. We then used FIMO (online server http://meme.nbcr.net/meme/cgi-bin/fimo.cgi) to scan the corresponding regions of all AS GYNNGYs, and determined whether a motif was statistically enriched in the strongly regulated group than the other regulated or unregulated group using the Fisher's exact test. We used *E-*value cutoff ≤1 x 10^-4^ in the FIMO scan. Using the cutoff ≤1 x 10^-3^ did not qualitatively affect our result.

### Evolutionary analyses

The non-synonymous (*d*_N_) and synonymous (*d*_S_) substitution rates between human and mouse protein-coding orthologs were extracted from Ensembl 71 by using BioMart (http://www.ensembl.org/biomart/martview/).

We measured the conservation of intronic sequences downstream of the GYNNGYs by using the PhastCons scores, downloaded from the UCSC database (22). The score was computed for each nucleotide site using the genome alignment of all sequenced placental mammals, and a higher score means higher conservation. To minimize errors, in this process we used only AS GYNNGYs which had at least one GY annotated as splice site by Ensembl.

To identify conserved AS GYNNGYs and AS NAGNAGs, we used the UCSC genome alignment chain files hg19ToMm10.over.chain and mm10ToHg19.over.chain as well as the program pslMap and our Perl program. Briefly, we first converted each splice site's coordinate into the UCSC PSL format and then found the orthologous region in target genome by coordinate mapping with pslMap. For example, we got orthologous regions in mice for human splice sites using this command: ‘pslMap –swapMap –chainMapFile human_splice_sites.psl hg19ToMm10.over.chain mouse_orthologous_region.psl’, where human_splice_sites.psl and mouse_orthologous_region.psl are the human splice sites and identified mouse orthologous regions, respectively. After an orthologous region for a splice site was identified, the information of the orthologous region was compiled, such as whether it was a splice site, and if so, what splicing status was. With these pieces of information, the conservation of a splice site could be estimated based on both splicing status and sequence identity. Since no chain files between mm10 and rheMac2 were available, the mouse sites were converted from mm10 to mm9 by using the same method and then converted to rheMac2. In the analysis of splice site gain and loss, we used one-to-one conserved regions across the three species.

### Analyzing the distribution of AS GYNNGYs across CDS and stop codons introduced by AS

To get the distribution of AS GYNNGY's occurrences along CDS, we selected the transcript with longest coding regions for each AS GYNNGY. Then the relative location was calculated as the distance of AS GYNNGY to the start codon divided by the total length of the CDS.

To check whether AS at GYNNGY could introduce stop codons in the downstream exons, we *in silico* translated the splicing isoforms produced by the AS event. If stop codons occurred in the immediate downstream exons, the event was regarded as stop-codon introducing.

## RESULTS

### Global analyses suggest splicing noise as the cause of AS at GYNNGY 5′ splice sites

We identified GYNNGY 5′ splice sites by mapping large-scale RNA-seq reads (16 human tissues from Illumina Body Map 2.0 and 9 mouse tissues from (23)) to human and mouse genomic sequences as well as gene models extracted from the databases Ensembl (21), UCSC (22) and NCBI (20) (Figure 1, and see Materials and Methods for details). In total, we identified 122 198 and 124 066 GYNNGY 5′ splice sites in humans and mice, respectively (Table 1, and see Supplementary Dataset S1 and S2 for the genomic locations of these AS sites). Among them, 796 (0.65%) and 1027 (0.83%) are alternatively spliced in humans and mice, respectively.

**Table 1.** Alternatively spliced GYNNGYs in protein-coding genes of humans and mice

	Human			Mouse		
	CDS	UTR	Total	CDS	UTR	Total
All GYNNGY	107 221	14 977	122 198	109 906	14 160	124 066
AS GYNNGY	621	175	796	845	182	1027
AS proportion	0.58%	1.17%	0.65%	0.77%	1.29%	0.83%
*P*-values*	< 2.2 x 10-16			3.861 x 10^-14^		

Each cell shows the counts or percentage of each type of GYNNGYs. CDS, protein-coding region; UTR: 5′ or 3′ untranslated regions. *, the *P-*values are for the chi-squared test of the proportions of AS GYNNGYs between CDS and UTRs.

The regulation model and the splicing noise model predict different outcomes in many aspects. First, the noise model predicts that AS at GYNNGYs may occur more often in untranslated regions (UTRs) than in coding regions (CDS), because AS in CDS would result in frameshift and deleterious effects if translated. The regulation model does not predict this tendency. In particular, if AS at GYNNGYs is mainly to increase proteome diversity, one would expect an opposite pattern—more AS GYNNGYs in CDS because AS only in CDS can change protein sequences. Our results show that UTRs have significantly higher proportions of AS GYNNGYs than CDS (Table 1, chi-squared test, UTR versus CDS: in humans, 1.17% versus 0.58%, *P* < 2.2 x 10^-16^; in mice, 1.29% versus 0.77%, *P* = 8.36 x 10^-11^), supporting the noise model.

**Figure 2.**
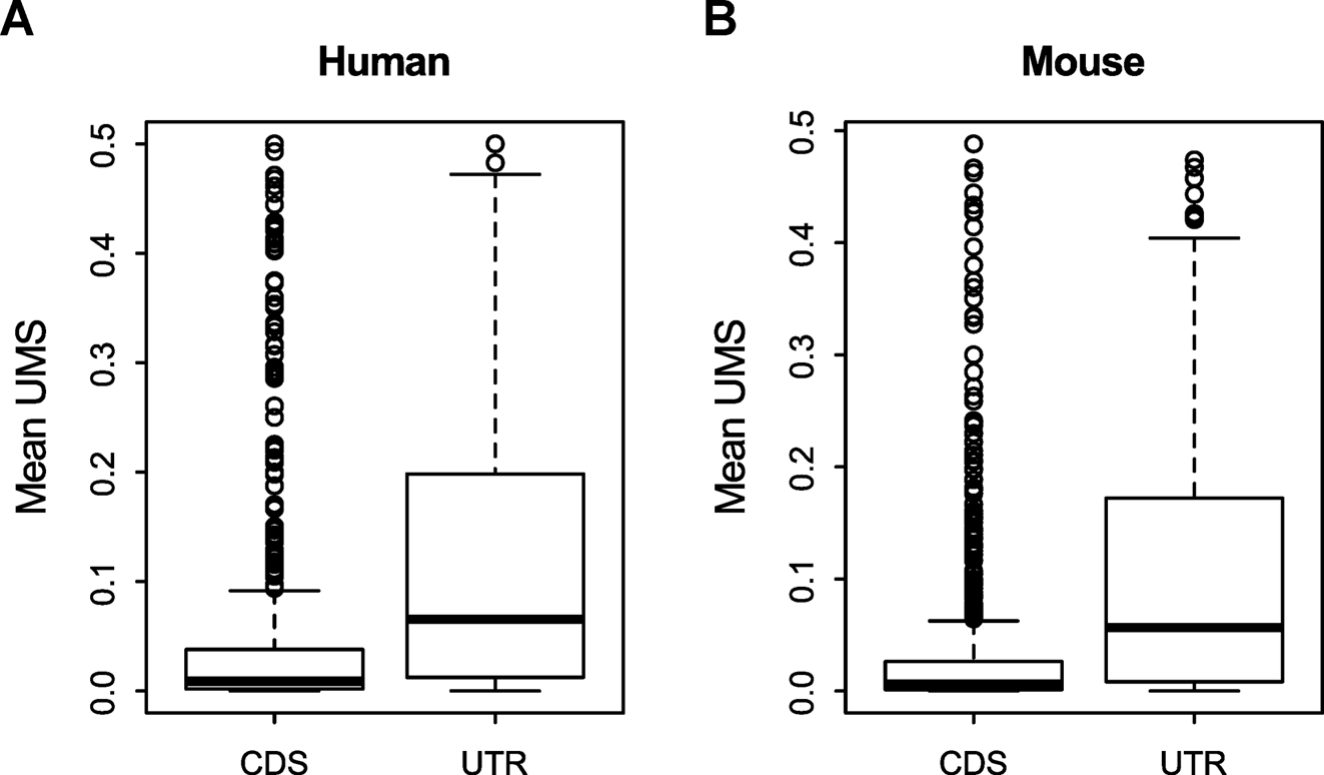
The UMS of AS GYNNGYs is significantly smaller in CDS than in UTR. Wilcoxon rank sum test: (A) human, *P* = 4.058 x 10^-16^, and (B) mouse, *P* < 2.2 x 10^-16^. The UMS is the mean across all examined tissues.

Second, the noise model regards the minor splicing form of each AS GYNNGYs as noise and predicts that their frequency should be lower in CDS than in UTRs, due to more deleterious effects in CDS as stated above. For each AS GYNNGY splice site, the minor splice site is defined as the one with lower average usage across all tissues. For convenience, we also define a variable UMS, which was calculated as the expression level of the minor splicing isoform divided by the expression of all the isoforms from the gene. The variable measures the relative abundance of the minor AS isoform for a gene in each tissue. As shown in Figure 2, the mean UMS across tissues in CDS is >6-fold lower than that in UTRs (Wilcoxon rank sum test: *P* = 4.058 x 10^-16^ and *P* < 2.2 x 10^-16^ for humans and mice, respectively), supporting the noise model. We reach the same conclusion when the maximum UMS across tissues is used (Supplementary Figure S1, *P* = 1.502 x 10^-11^ and 6.252 x 10^-16^ for humans and mice, respectively).

**Figure 3.**
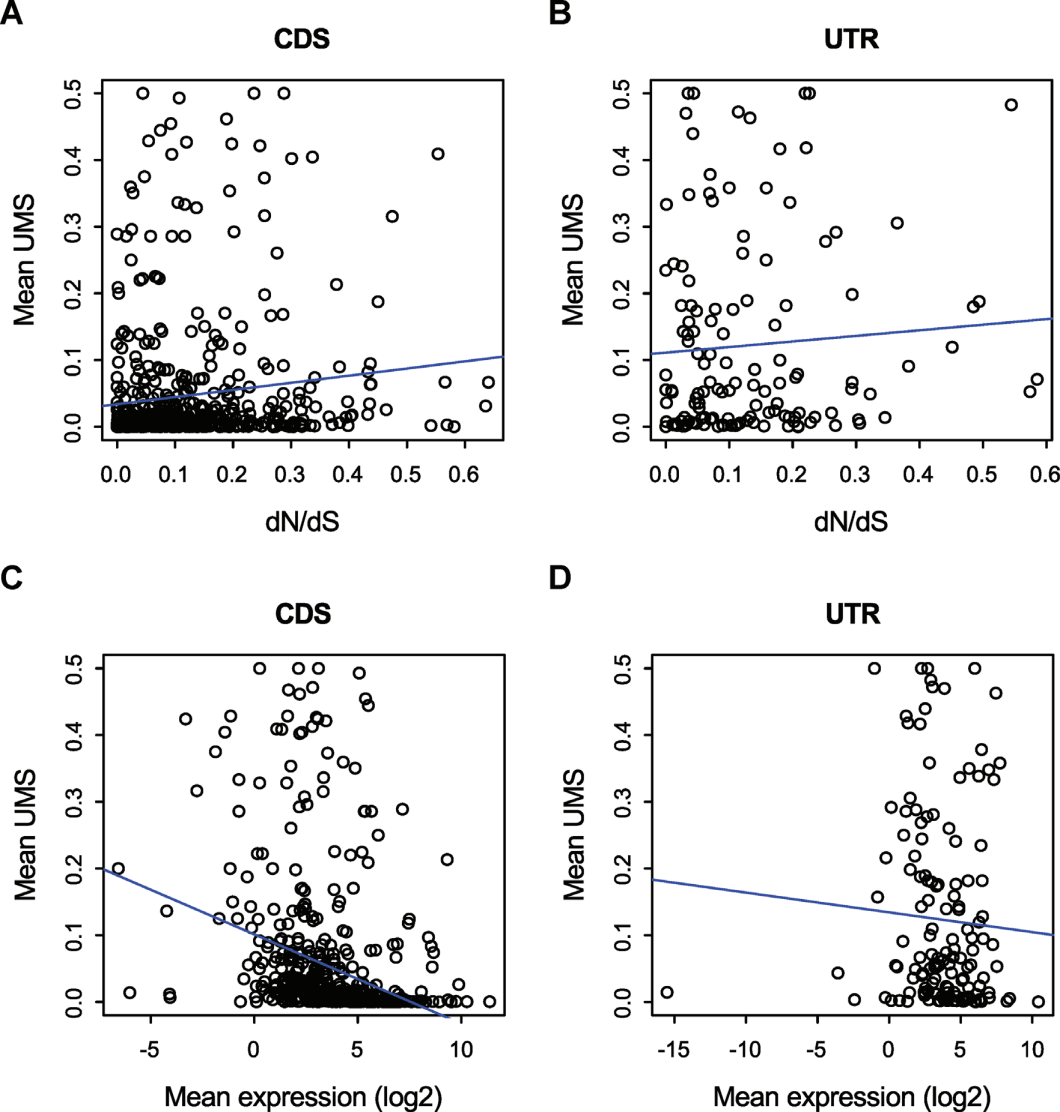
The UMS of AS GYNNGYs in CDS is positively correlated with the ratio of non-synonymous (*d*_N_) and synonymous (*d*_S_) substitution rates and negatively correlated with gene expression levels (panels A and C, Spearman's rho = 0.18, *P* = 1.95 x 10^-5^ and rho = −0.51, *P* < 2.2 x 10^-16^, respectively). The same trends are shown for UTR, too, but only significant for the case of gene expression (panels B and D, rho = 0.09, *P* = 0.31 and rho = −0.17, *P* = 0.0393, respectively).

Third, the noise model also predicts that the UMS at AS GYNNGYs is lower in more conserved genes and in more highly expressed genes, because these kinds of genes are subject to stronger functional constraints (29) and thus may be less tolerable to noises. The regulation model may predict that UMS of AS GYNNGYs is comparable among these genes because there is no reason to argue why regulation is less needed for highly expressed or conserved genes. Again, our data support the noise model: the UMS in CDS is positively correlated with protein conservation level (measured as the ratio of non-synonymous to synonymous substitution rates) (Figure 3A and Supplementary Figure S2A, Spearman's Rho = 0.18, *P* = 1.95 x 10^-5^ for humans, and Rho = 0.12, *P* = 1.23 x 10^-3^ for mice), and is negatively correlated with mean gene expression level (Figure 3C and Supplementary Figure S2C, Rho = −0.51, *P* = <2.2 x 10^-16^, and Rho = −0.49, *P* < 2.2 x 10^-16^, for humans and mice, respectively). The results are confirmed when analyzing each tissue's expression data separately (Supplementary Table S3). Intriguingly, much weaker but significant negative correlations between UMS and gene expression levels are also detected in UTRs (Figure 3D and Supplementary Figure S2D, Spearman's Rho = –0.17, *P* = 0.04, and Rho = –0.20, *P* = 0.01 for humans and mice, respectively), suggesting that abundant noises in UTRs may also be deleterious and intolerable. No significant correlation is observed between UMS and protein conservation level in UTR (Figure 3B and Supplementary Figure S2B, *P* > 0.1).

In summary, our global analyses suggest that AS at GYNNGY 5′ splice sites may be caused by splicing noise.

### Approximately 20% and 3% of AS GYNNGYs in humans and mice show tissue-specific regulation

Our above conclusion does not rule out that some AS GYNNGY events may be results of gene regulation. The ideal way to distinguish noisy AS and regulatory one may be examining the effect of impairing the minor isoform of a given AS GYNNGY. If it is functional, it would result in some phenotypic changes. Unfortunately, this approach is impractical in genome-scale analysis at present and no such large-scale data exist for us to test each AS GYNNGY. Alternatively, we identified tissue-dependent AS events, because regulatory AS events often provide tissue-specific functions by varying abundance of splicing isoforms (30). We use this feature as a proxy of an AS event being functional (also see Discussion).

Following this strategy, we evaluated the variation of UMS for each AS GYNNGY across 16 human tissues. Briefly, we used permutated chi-squared tests to detect AS events that deviated from the null hypothesis that the UMS was constant in all the tissues where the gene was expressed. The tests required that an AS GYNNGY has sufficient RNA-seq reads from at least two tissues (see Materials and Methods), so 621 GYNNGYs in humans and 738 in mice met this requirement and remained for tests. We also measured the variation magnitude using ΔUMS, calculated as the absolute difference between the maximum and the minimum UMS across tissues. ΔUMS ranges from 0 to 1 and is related to ΔPSI (16), but ΔUMS is more convenient for us to get the variation of minor splicing isoforms. In total, we identified 277 (45%) of 621 testable human AS GYNNGYs showing tissue-dependent regulation (Table 2, FDR < 0.01). Among them, 118 (19%, i.e. 118/621) have ΔUMS ≥ 0.25, so marked as strongly regulated. For comparison, we further divide the regulated sites with ΔUMS < 0.25 into ‘medium’ (0.1 ≤ ΔUMS < 0.25), ‘weak’ (0.05 ≤ ΔUMS < 0.1) and ‘very weak’ (ΔUMS < 0.05), and regard those AS GYNNGYs with FDR > 0.01 as ‘unregulated’. In the same way, 50 of 738 (6.8%) testable mouse AS GYNNGYs are under tissue-dependent regulation (FDR < 0.01) and 21 (2.9%) are strongly regulated (Table 2).

**Table 2.** Alternatively spliced GYNNGY sites showing tissue-dependent regulation

	Human			Mouse		
	CDS	UTR	Total	CDS	UTR	Total
Total sites^a^	510	111	621	652	86	738
Regulated^b^	219	58	277	41	9	50
Strongly regulated (%)^c^	75 (14.71)	43 (38.74)	118 (19.00)	15 (2.30)	6 (6.98)	21 (2.85)

^a^Only sites that had expression in at least two tissues (≥10 reads in each tissue) are considered, because at least two tissues are needed for chi-squared test.

^b^The sites that showed tissue-specific regulation (FDR ≤ 0.01).

^c^The regulated sites that had ΔUMS ≥ 0.25. The proportion of strongly regulated GYNNGYs is significantly higher in UTRs than in CDS (chi-square test, *P* = 1.094 x 10^-8^ for human and Fisher's exact test, *P* = 0.02689 for mouse).

One possible caveat for getting above results is that some tissues might be more tolerable to splicing noise or tissue-specific splicing factors could promote noisy splicing, leading to upregulation of noisy splicing isoforms in only some tissues. If this is true, one expects most AS GYNNGYs show maximal UMS in the same set of tissues. Our data show that the maximal UMSs are nearly evenly distributed for most tissues (Supplementary Figure S3), rejecting this hypothesis.

As the number of identified AS GYNNGYs depends on the number of sampled tissues, we inferred the upper bound for proportion of strongly regulated GYNNGYs in an organism by extrapolation (see Materials and Methods). In humans, the inferred proportion of strongly regulated GYNNGYs is 20% (Supplementary Figure S4). In mice, we could not make a reliable inference due to too few tissues. However, the upper bound for mouse is probably smaller than human's, because the proportion of mouse-regulated AS GYNNGYs is always smaller than human's when the same number of tissues are considered (Supplementary Figure S4). Therefore, the proportion of strongly regulated AS GYNNGYs in mammals may be up to 20%.

Interestingly, the proportion of strongly regulated AS GYNNGYs is significantly higher in UTR than in CDS (Table 2, *P* < 0.05). This result suggests that AS GYNNGYs in UTRs are more likely functional, and also raises a possibility that the higher proportions of AS GYNNGYs in UTR than in CDS observed at the beginning (Table 1) may be due to larger proportions of regulatory sites rather than weaker deleterious effects in UTRs. To test this, we compared the proportions of AS GYNNGYs between UTR and CDS after eliminating the strongly regulated sites. The proportions are slightly changed and remain significantly higher in UTR than in CDS (Supplementary Table S4, chi-squared test: *P* = 1.146 x 10^-8^ and 4.032 x 10^-13^ for humans and mice, respectively). These results suggest that both weaker deleterious effects and higher frequency of regulation contribute to higher proportions of AS GYNNGYs in UTRs, but the former is predominant.

### The regulated GYNNGYs are more conserved than the unregulated ones

To gain more confidence on the regulated GYNNGYs being functional, we investigated the conservation of the splicing status of GYNNGYs, because conservation on features implies functions (31).

We regard an AS GYNNGY conserved only when it is alternatively spliced in both humans and mice. We found that in CDS the strongly regulated GYNNGYs are more conserved than the other less regulated GYNNGYs (Table 3, *P* = 1.31 x 10^-4^ and *P* = 0.013 for humans and mice, respectively), indicating that AS events at the regulated GYNNGYs are subject to stronger functional constraint than other events. Also note that the (very) weakly regulated and unregulated sites have similar conservation levels, suggesting that the (very) weakly regulated sites may have insignificant functions and/or are recently evolved.

**Table 3.** Conservation of splicing status for alternatively spliced GYNNGYs between human and mouse

		Strong	Medium	Weak	Very weak	Unregulated
Human	Conserved	26	16	5	3	43
	Total	73	64	38	41	279
	Conserved proportion	35.62%	25.00%	13.16%	7.32%	15.41%
Mouse	Conserved	8	14	9	9	47
	Total	25	52	50	59	456
	Conserved proportion	32%	26.92%	18.00%	15.25%	10.31%

The groups of AS GYNNGYs are as follows: strong, ΔUMS ≥ 0.25 and FDR ≤ 0.01; medium, 0.1 ≤ ΔUMS < 0.25 and FDR ≤ 0.01; weak, 0.05 ≤ ΔUMS < 0.1 and FDR ≤ 0.01; very weak, ΔUMS < 0.05 and FDR ≤ 0.01; unregulated, FDR > 0.01. In mouse, we used FDR ≤ 0.1 as cutoff for regulated sites because FDR ≤ 0.01 would lead to too few sites for reliable test. The strongly regulated AS GYNNGYs is more conserved than the other groups (Fisher's exact test, *P* = 0.0001311 and *P* = 0.01262 for humans and mice, respectively).

The accuracy of conservation analysis heavily depends on the powers of detecting AS events, which is influenced both by the number of examined tissues (Supplementary Figure S4) and by the sequencing coverage (see Discussion). It is unknown whether this issue would have biased the comparison between strongly and weakly regulated GYNNGYs. To avoid this problem, we also evaluated AS conservation at sequence level, that is, we regard a human AS GYNNGY conserved in mice if the mouse orthologous splice site is identical to that of humans, regardless of the splicing status determined by RNA-seq. This method confirms our results based on splicing status (Supplementary Table S5, *P* = 0.005 and 0.14 for humans and mice, respectively), though the mouse data is not statistically significant, perhaps due to the smaller sample size.

### The regulated GYNNGYs are flanked by distinctive sequence features

What is the mechanism for regulated AS at GYNNGYs? In general, both tissue-dependent *trans*-regulators (e.g. splicing factors) and *cis*-elements (e.g. sequence motifs) control tissue-dependent regulation (30). However, currently no large-scale data are available for tissue-specific splicing regulators and their target genes, preventing us linking the regulation of AS GYNNGYs to tissue-specific splicing factors and targeting motifs. Instead, we focus on identifying *cis*-features that may contribute to tissue-dependent regulation (30).

**Figure 4.**
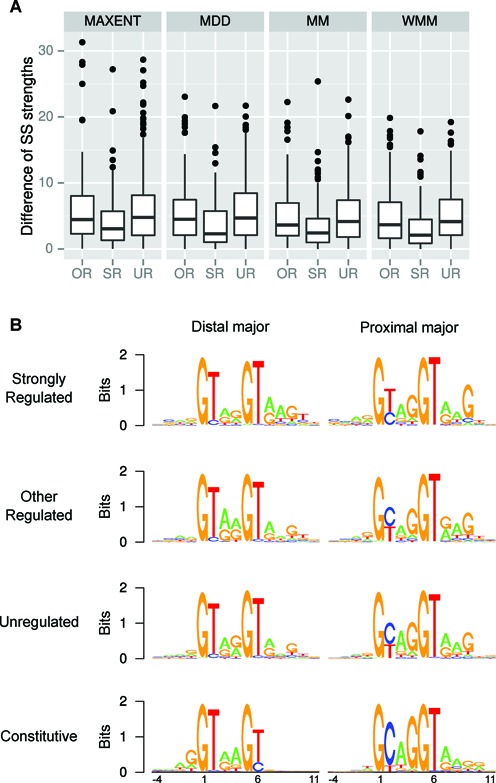
The two tandem splice sites in each strongly regulated GYNNGY are more similar to each other than those in GYNNGYs under weaker or unregulation. (A) Comparison of absolute splice score (SS) differences among different groups. SR, strongly regulated; OR, other less regulated; UR, unregulated. Splice site scores are measured with four different methods (‘MAXENT’, ‘MDD’, ‘MM’ and ‘WMM’). All comparisons are significant (Wilcoxon rank sum test, all *P* < 0.001). (B) The consensus sequences of GYNNGY splice sites. For each group, the GYNNGYs are further divided into two: one mainly using the first GY (distal major) and one mainly using the second GY (proximal major).

First, we found that the splice score difference of the two tandem alternative 5′ splice sites in each regulated GYNNGY is averagely smaller than that in less or unregulated GYNNGYs (Figure 4A, *P* < 0.001), and magnitudes of the differences are negatively correlated with the degree of regulation (measured by ΔUMS) (Supplementary Figure S5). This result confirms the idea that functional AS tandem splice sites often have two similar splice sites and the relative strengths of two splice sites in a tandem splice site are the major determinant of splicing, which was advocated by studies of AS NAGNAG 3′ splice sites (16).

Second, we examined the consensus sequences of splice sites in each group of GYNNGYs (Figure 4B). As expected from the above result, the two tandem 5′ splice sites in each of strongly regulated AS GYNNGYs are similar, matching the consensus of 5′ splice site quite well (Figure 4B). In contrast, the two tandem splice sites in each of other regulated sites and in the unregulated ones are more different. The most conspicuous is: when the first GY is GT, splicing would probably occur there, otherwise it occurs at the second GY. This suggests that the first GY is preferred to the second and that the nucleotides at these two positions are very crucial in choosing splice site.

**Figure 5.**
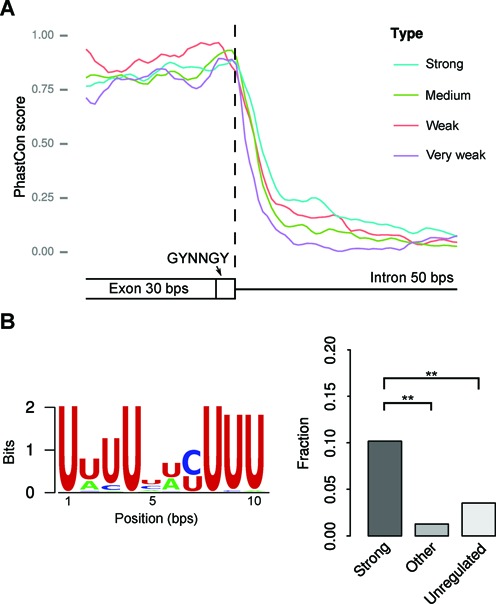
(A) Strongly regulated AS GYNNGYs have more conserved downstream flanking intronic sequence than other AS GYNNGYs (paired Wilcoxon rank sum test, *P* < 1 x 10^-6^). The vertical dash line indicates the end of GYNNGY. (B) The U-rich motif is enriched in strongly regulated AS GYNNGYs (**P < 0.01). ‘Strong’, ‘Medium’, ‘Weak’ and ‘Very weak’ denote GYNNGY groups with different degrees of regulation (measured by ΔUMS). ‘Other’ consists of ‘Medium’, ‘Weak’ and ‘Very weak’ groups. ‘Unregulated’ represent unregulated AS GYNNGYs.

Third, we found that the 50 nucleotides downstream of strongly regulated GYNNGY are more conserved than that of other less regulated GYNNGYs (Figure 5A, *P* < 1 x 10^-6^), similar to the case of NAGNAG 3′ splice sites (16). This indicates that the sequence regions downstream of regulated GYNNGYs may contain some regulatory motifs. We used the software MEME to search motifs that are enriched in the downstream 50 nt flanking intronic regions of strongly regulated GYNNGYs (see Supplementary Table S6 for all identified motifs). One U-rich motif (Figure 5B) shows significant enrichment in the regions of the strongly regulated GYNNGYs than in the regions of less or un- regulated GYNNGYs (Supplementary Table S6, Fisher's exact test, *P* < 0.01). U-rich motifs were reportedly enriched in the downstream flanking introns of alternative cassette exons and promoting inclusion of cassette exons (32). Our results suggest that this motif may also be involved in regulation of alternative 5′ splice sites. A few other motifs are also slightly enriched in strongly regulated AS GYNNGYs when a stricter criterion (*E*-value < 1 x 10^-4^) for motif search is chosen (Supplementary Tale S6). These motifs are trivial for explaining all the regulated GYNNGYs, but could still be useful to study regulation of associated splice sites in future.

### Regulated GYNNGYs may mainly regulate gene expression levels

A remaining question is how regulated GYNNGYs contribute to functions. An expected effect of AS GYNNGYs in CDS is frameshift, which in turn leads to dramatic change of protein sequence or introducing premature stop codon followed by nonsense-mediated mRNA decay (NMD). If increasing proteome diversity is the main role of AS GYNNGYs, then the former effect should be predominant.

**Figure 6.**
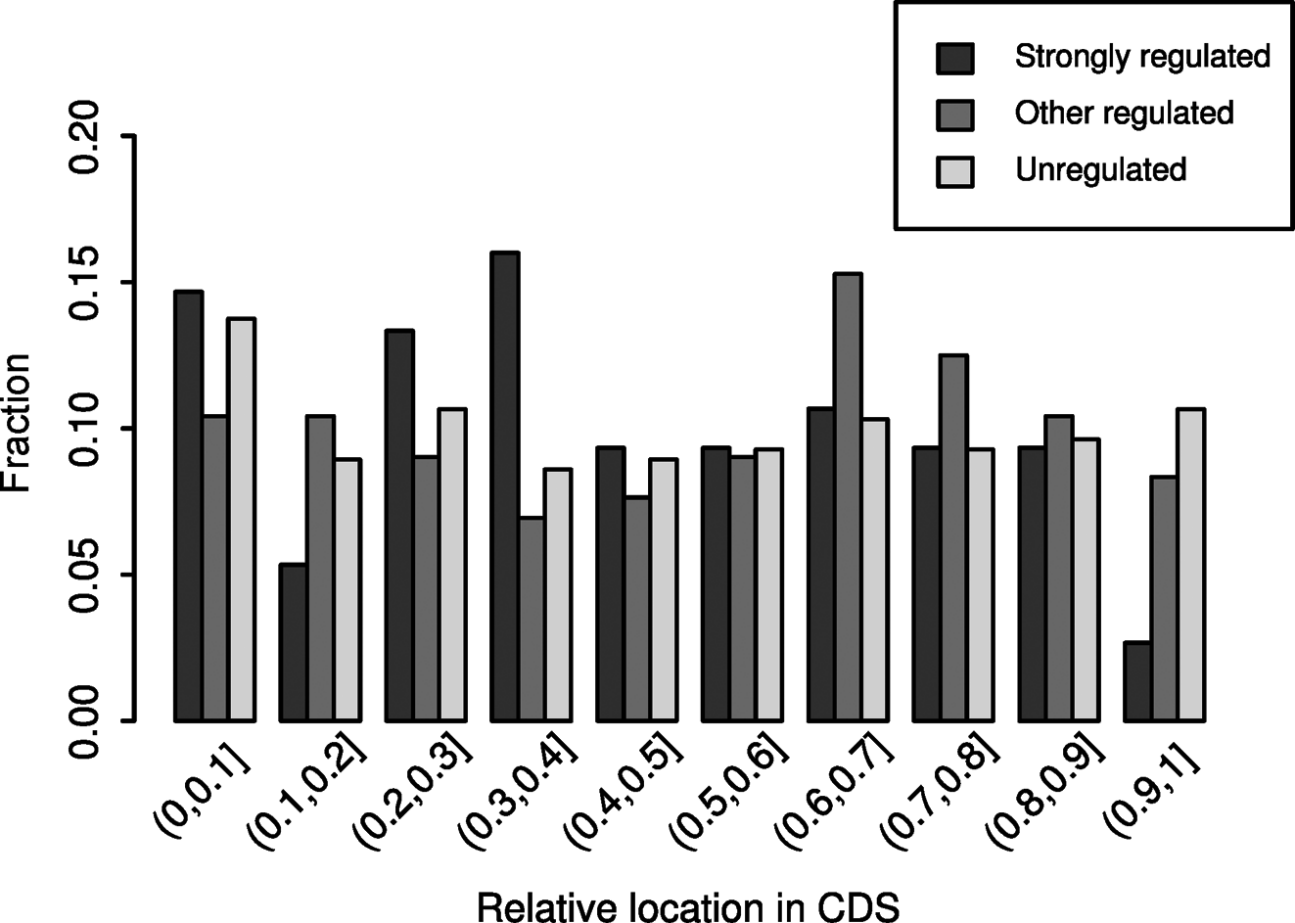
The distributions of AS GYNNGYs along CDS. The distribution of strongly regulated sites is largely similar to those of other sites (one-sided Kolmogorov–Smirnov test, *P* = 0.056 and 0.19 when compared to more weakly regulated or unregulated sites, respectively). CDS is divided into 10 bins of equal length for each gene.

First, we found that 72% of strongly regulated AS GYNNGYs introduce stop codons immediately in the downstream exons, and the proportions do not differ significantly among strongly regulated GYNNGYs and other groups (Table 4, Fisher's exact test, *P* > 0.74). Previously, 78% of AS GYNNGYs were reported to induce stop codon located more than 50 nucleotides upstream of the last exon-exon junction (19). The slight decrease of the proportion in our study may result from using different gene models (Ensembl instead of NCBI Refseq) and from examining only one downstream exon (premature stop codons may appear in further downstream exons). Second, we examined the distribution of AS GYNNGYs across CDS length. We found that AS at the strongly regulated GYNNGYs occur evenly along CDS and do not significantly differ from other regulated and unregulated sites (Figure 6, one-sided Kolmogorov–Smirnov test, *P* > 0.05). These two results indicate that most of the regulated GYNNGYs may introduce premature stop codons and trigger NMD to decay the splicing isoforms. In this way, gene expression levels can be regulated (33). For the cases that do not introduce immediate stop codons, the changes of proteins can be large (>10 AAs) (Table 4). These results suggest that the roles of regulated GYNNGYs are primarily to regulate gene expression levels and secondarily to encode different proteins.

**Table 4.** The effect of AS GYNNGY on protein sequences

	Total	Change < 10 AAs^b^	Change >10 AAs^b^	Frameshift	Stop (%)^c^
Strongly regulated^a^	75	0 (0%)	1	74	54(72%)
Other regulated^a^	144	5 (3.5%)	3	136	107(74%)
Unregulated^a^	291	7 (2.4)	5	279	216(74%)

^a^The grouping of sites is as defined in the text, ‘strongly regulated’ sites have ΔUMS ≥ 0.25 and ‘other regulated’ consists of all other regulated sites.

^b^These two types of changes occur when the translational frame is recovered to the original one through AS at the downstream acceptor site (the number of changed amino acids (AAs) are recorded).

^c^The number of cases introducing stop codons in the immediate downstream exon. No difference exists between these three groups of sites (Fisher's exact test, all *P*-values > 0.1).

## DISCUSSION

### Gene regulation or splicing noises?

On one hand, AS can provide gene regulation in diverse ways, such as encoding different protein isoforms and tuning mRNA expression (4,33,34). On the other hand, splicing noise can also lead to AS (35,36). Our results, together with previous studies on NAGNAG 3′ splice sites (15,16), support that both gene regulation and splicing noise account for AS at tandem splice sites, and splicing noise is probably predominant.

At first glance, it may be hard to imagine why cells produce something useless or even bad. However, it becomes understandable after we know that genome and its regulation are not perfect (37,38). In RNA splicing, general splicing factors find the splice sites in a tentative way (12). When two splice sites are near, it is unlikely for splicing factors only bind one splice site but not the other, unless other specific factors interfere. Based on this idea, a stochastic model (15,16) has been proposed, stating that the usages of two nearby splice sites are proportional to their relative binding strengths by splicing factors. The validity of the model is well supported by high accuracy of predicting usage of splice sites at NAGNAG 3′ splice sites (15,16). Based on this model, noisy splicing may be inevitable if one new splice site appears near a functional one. On the other hand, an evolutionary model of alternative 5′ and 3′ splice sites (13) proposed that mutations near original splice sites could create new splice sites, leading to alternative 5′ and 3′ splice sites (13). Combining this evolutionary model and the stochastic model, one can imagine that newly evolved splice sites compete with the original one, no matter they are functional or not. Probably newly evolved splice sites initially have no functions but competing with functional sites. This would result in many events of noisy splicing. Some, likely a minority, of these new sites would eventually evolve new functions. This explanation predicts that older (thus more conserved) AS splice sites are more likely functional, just as we observed (Table 3). In addition, some noisy splice sites may be fixed through neutral evolution as long as the usage of the noisy site is low and thus has little effect on organismal fitness. This hypothesis predicts that fixed noisy splice sites are usually weaker than functional ones in order to minimize the noise. Our data support this prediction, showing that compared to minor splice sites in strongly regulated AS GYNNGYs (more likely functional), those in less regulated AS GYNNGYs are much less competitive to the major splice sites (Supplementary Figure S5), and that the usage of minor splice sites is significantly higher in strongly regulated AS GYNNGYs than in weakly regulated or unregulated sites (Supplementary Table S7). In sum, both inherent stochasticity of splicing process and evolutionary process of new splice sites predetermine occurrences of splicing noise.

Now the challenge is to distinguish functional AS events from noisy ones. AS regulates genes in multiple ways, such as encoding different proteins (4), tuning gene expression levels by modifying miRNA target sites (34) and changing translational efficiency, but identifying functional AS events using genomic approach is not simple (31,39). In this study, as a previous study did (16), we regard tissue-regulated AS events as functional events. This strategy can identify AS events with different isoforms needed in different tissues, but may leave out functional AS events that have constant splicing isoform ratio among tissues. Actually, the relationship between splicing variation among tissues and functions has been discussed by Hiller and Platzer (40), but it should not significantly affect our results for the following reasons. First, the functional AS events with constant splicing ratios cited by that paper actually have high expression for all splicing isoforms (e.g. the ratio of the two splicing isoforms of *WT1* is 55%:45% (41)), different from the very low expression of minor splicing isoforms in our unregulated AS events (most have UMS <5%, Supplementary Table S8). Second, many cited functional AS events claiming for constant splicing ratios obviously have varying splicing ratio among examined tissues, such as *FGFR1* (42) and *ING4* (43), supporting that functional events usually vary splicing ratios. Third, our unregulated AS events are less conserved than the regulated ones (Table 3), suggesting that they are less likely to function. These arguments support that the approach of identifying functional AS GYNNGY events by using tissue-dependent regulation is generally reliable and may suffer a low rate of false negatives.

Using this strategy, we identified ∼20% and ∼3% of AS GYNNGYs under strong tissue-specific regulation (ΔUMS ≥ 0.25). Here, the proportion of identified human-regulated GYNNGYs is smaller than that reported for all alternative 5′ splice sites (5) (20% versus 64%), even if more weakly regulated ones are counted. There are multiple possible reasons for this discrepancy. First, GYNNGYs may *bona fide* have smaller proportion of regulated sites than the average of all alternative 5′ splice sites. Second, it is caused by smaller number of tissues used by that study (10 versus 16 tissues). Third, and more importantly, it may be due to different sequencing coverages (Supplementary Table S1). In that study, a tissue has less than 29 million reads, much smaller that the 73 million or more reads used in this study. Moreover, the reads there are 32 nt long and single-end, which are much less effective when mapped onto genome. These factors lead to a much lower sequencing coverage in that study than in our study, and thus to lower power to detect noisy isoforms which often have very low expression. The effect of sequencing coverage on capacity to detect noisy isoforms is supported by comparing the numbers of identified AS GYNNGYs in rhesus monkey and in mouse in our data set. The former has longer and more reads per tissue than the latter, and it also detected much more noisy AS GYNNGYs (Supplementary Table S1). These results indicate that sequencing coverage may be one major determinant of detecting low-level splicing isoforms. Therefore, with increasing sequencing coverage, we may find even higher proportion of noisy splicing events.

These proportions of regulated events are also smaller than those of AS NAGNAG 4′ splice sites (37% and 12% for humans and mice, respectively) (16), indicating that AS GYNNGYs may be more often caused by noise than AS NAGNAGs are. At first sight, this result may be surprising because one may expect a lower proportion of noisy events for AS GYNNGYs due to more deleterious effect (frameshifts) than AS NAGNAGs (primarily affecting one or two amino acids). Why do we observe an opposite pattern? Note that the deleterious effect of a noisy splicing event depends not only on the caused structural change, but also on the splicing isoform's abundance. Noisy isoforms at GYNNGYs may have very low expression level to minimize deleterious effect. This hypothesis is supported by the lower UMS values in unregulated AS GYNNGYs than in unregulated AS NAGNAGs (Supplementary Table S7, *P* = 6.07 x 10^-21^). On the other hand, a new splicing isoform from the AS of NAGNAG may be easier to evolve functions for unknown reasons. This would be true if evolution of novel functions is more often by incorporating mild-effect mutations than strong ones.

All these proportions of regulated splice sites (both GYNNGYs and NAGNAGs) are based on detected AS events only. As argued above, leaving out low-frequency AS events may underestimate the proportion of noisy splicing events, and this effect may be stronger for GYNNGYs than for NAGNAGs because of its averagely lower expression. In another word, the proportions of functional AS events in GYNNGYs will be even lower than in NAGNAGs when the power of detecting low-frequency splicing isoforms is increased. Considering this issue, we reason that the proportions of functional AS NAGNAGs by a previous study (16) may be overestimated, because AS events that did not have all splicing isoforms expressed at PSI > 5% in at least one tissue were excluded, which are more likely splicing noise. Reanalyzing the same data set confirms this suspicion (Supplementary Table S9). We also found that the gain and loss of AS status during evolution are more often for GYNNGYs than NAGNAGs (Supplementary Table S10), implying weaker functional constraints on AS events at GYNNGYs.

Interestingly, humans have a much larger proportion of strongly regulated AS GYNNGYs than mice (∼20% versus ∼3%). A similar pattern (37% versus 12%) was observed for AS NAGNAG 3′ splice sites (16). Does this observation mean humans take advantage of splicing regulation more often than mice? Actually, technical factors, such as sequencing coverage and sample processing protocols, can contribute to this difference between humans and mice. On the other hand, more diverse genetic backgrounds in human samples may be another important contributor. The human samples include many individuals with different genders, a range of ages and even from different populations (Supplementary Table S1), while the mouse tissues were from only one individual. Studies have reported that genders (44), ages (45) and population ancestries (46) all contribute to variation of AS. Therefore, possibly these factors all have contributed to the proportion difference between humans and mice. Population ancestry is less likely to explain the difference because all but one tissue come from the same population (Supplementary Table S1). We roughly estimated how much gender and age have contributed to the occurrences of regulated GYNNGYs by employing the results from previous studies (5,19,44,45). Assuming that the number of AS events linearly increases with the number of examined tissues, we estimated that gender may account for 10.5 regulated AS GYNNGYs in 16 tissues (Supplementary Table S11). However, this number may be an underestimate because 12 tissues used in the study (44) are all from human brain and thus represent lower diversity. Interestingly, we found that age may account for 236.5 regulated AS GYNNGYs based on the data of human brain development (45). This number is very close to the number of regulated AS GYNNGYs in our study (236.5 versus 277, see Supplementary Tables S11 and S2) and much larger than that of strongly regulated ones (236.5 versus 118), suggesting that the large range of ages in human samples might explain most part of the difference of the proportions between humans and mice. However, our estimates are very rough, and probably are overestimates because our assumption that the number of splicing events linearly increases with examined tissue number is not true (see Supplementary Figure S4). Moreover, experiments of studying splicing in a few genes individually suggest that age has a much smaller contribution to splicing variation than tissue types do (47). Therefore, the human-regulated AS GYNNGYs possibly arise mainly from different tissue types and thus the proportion difference between human and mice suggests more regulation on human than on mouse GYNNGY 5′ splice sites. One open question is what the relative contributions of different biological factors are, including tissue types, ages, genders and populations, which could be studied in future using the same sequencing data in order to controlling for confounding factors, such as ways of RNA sample processing, sequencing coverage, etc.

### The roles of regulated AS GYNNGYs

It is straightforward to imagine that a regulated AS GYNNGY in CDS can produce different protein isoforms because of frameshifts. Among the regulated GYNNGYs, we found that only ≤3.5% of AS events have translational frame recovered by downstream AS within 10 amino acid distance (Table 4). For the remaining, most of them (>72%) actually introduce stop codons in the adjacent downstream introns. Since these premature stop codons may often trigger NMD, the primary role of these events may tune gene expression rather than encode different proteins. This is different from the roles of AS at 3′ splice sites NAGNAGs, where the AS often causes deletion, insertion or modification of one or two amino acid (16). The role of AS in regulating gene expression by coupling with NMD has been evidenced by studies on specific genes (33) as well as by transcriptome data of mouse development (30). Our results suggest that the coupling between AS and NMD also regulates expression in a tissue-dependent manner.

In addition, AS can also change gene expression by coordinating with other processes, such as modifying miRNA target sites or translation-related motifs (48). One example is the AS GYNNGY in the gene *NDUFS5*, which occurs 2 nt upstream of the start codon (49). The AS results in two splicing isoforms, NM_004552 and NM_001184979, and NM_004552 has four more nucleotides ‘GTAG’ before start codon, giving a different kozak sequence. By checking the ribosome binding data (50) and gene expression data for *NDUFS5* in HEK293 cells line, we found that the translational efficiency of NM_004552 is ≥3 times stronger than that of NM_001184979 (Supplementary Table S12).

As we stated, tissue-dependent regulation is only a signal that an AS event is functional. To reveal its impact on phenotypes and fitness, one needs to examine the effects at cellular and organismal levels by impairing each AS event. This remains a challenge for genomic studies, but new technologies, such as the CRISPR/Cas system (51), seems shedding light on this goal.

In conclusion, by studying AS at GYNNGY 5′ splice sites, we propose that primarily splicing noise and secondarily gene regulation contribute to the occurrences of AS at tandem splice sites, and the reported proportion of splicing noise may increase when sequencing coverage increases. Our results also emphasize that one need distinguish functional and non-functional AS events when using genomic approaches; this principle may also apply to genomic studies of other features (31).

## SUPPLEMENTARY DATA

Supplementary Data are available at NAR Online.

SUPPLEMENTARY DATA
