# Supplementary material for: Alternative splicing at GYNNGY 5′ splice sites: more noise, less regulation
Source: Nucleic Acids Res. 2014 Nov 26;42(22):13969–80. doi: 10.1093/nar/gku1253 (PMC4267661; doi:10.1093/nar/gku1253)
Supplement: SUPPLEMENTARY DATA [file supp_42_22_13969__index.html]

Alternative splicing at GYNNGY 5′ splice sites: more noise, less regulation — Alternative splicing at GYNNGY 5′ splice sites: more noise, less regulation — SUPPLEMENTARY DATA 

# Alternative splicing at GYNNGY 5′ splice sites: more noise, less regulation

## SUPPLEMENTARY DATA

**Files in this Data Supplement:**

- SUPPLEMENTARY DATA
