# Supplementary material for: Alternative splicing at GYNNGY 5′ splice sites: more noise, less regulation
Source: Nucleic Acids Res. 2014 Nov 26;42(22):13969–80. doi: 10.1093/nar/gku1253 (PMC4267661; doi:10.1093/nar/gku1253)
Supplement: SUPPLEMENTARY DATA [file supp_gku1253_nar-02514-z-2014-File008.zip › Supplementary materials.pdf]

Title: Alternative splicing at GYNNGY 5' splice sites: more noise, less regulation

Supplementary materials

Supplementary materials 1
Supplementary Methods 2
Supplementary Tables 3
Supplementary Datasets (see separate files) 13
Supplementary Figures 13

Supplementary Methods

Estimate the translational efficiency of the gene *NDUFS5*

The gene *NDUFS5* has two splicing isoforms NM_004552 and NM_001184979. We got the frequency of ribosome binding at a translation initiation site in HEK293 cells using the high-throughput data of translation initiation sites sequencing (TIS-seq) (50), which is proportional to the number of transcripts under translation. The study used two different inhibitors (LTM and CHX) to block ribosomes to the start codon. We obtained the RNA-seq reads in HEK293 cells from GEO dataset GSE52530, of which two samples GSM1269354 and GSM1269355 were used. Mapped reads to the two splicing isoforms were determined by bowtie (parameters: ≤ 2 mismatches and at most 2 good hits -v 2 -k 2). The translational efficiency is calculated as the ratio of unique TIS-seq reads to unique RNA-seq reads for a given transcript.

Supplementary Tables

Table S1. RNA-seq samples used in this study

Organism	Read type (PE, paired-end)	Sequencing strategy	Platform	Read counts	Tissue	# AS GYNNGYs (# strongly regulated)	GEO accession	Gender	Population/Strain	Age
Human	2x50 PE	ploy(A)-selected, un-stranded	Illumina HiSeq 2000	77300072	adipose	796 (118)	GSM759491	female	Caucasian	73
	2x50 PE	ploy(A)-selected, un-stranded	Illumina HiSeq 2000	74472871	adrenal		GSM759493	male	Caucasian	60
	2x50 PE	ploy(A)-selected, un-stranded	Illumina HiSeq 2000	73513047	brain		GSM759495	female	Caucasian	77
	2x50 PE	ploy(A)-selected, un-stranded	Illumina HiSeq 2000	75862215	breast		GSM759497	female	Caucasian	29
	2x50 PE	ploy(A)-selected, un-stranded	Illumina HiSeq 2000	82437443	colon		GSM759499	female	Caucasian	68
	2x50 PE	ploy(A)-selected, un-stranded	Illumina HiSeq 2000	82918784	heart		GSM759501	male	Caucasian	77
	2x50 PE	ploy(A)-selected, un-stranded	Illumina HiSeq 2000	80397337	kidney		GSM759503	female	Caucasian	60
	2x50 PE	ploy(A)-selected, un-stranded	Illumina HiSeq 2000	81217148	leukocyte		GSM759521	male	Caucasian	58
	2x50 PE	ploy(A)-selected, un-stranded	Illumina HiSeq 2000	80048623	liver		GSM759505	male	Caucasian	37
	2x50 PE	ploy(A)-selected, un-stranded	Illumina HiSeq 2000	79296905	lung		GSM759507	male	Caucasian	65
	2x50 PE	ploy(A)-selected, un-stranded	Illumina HiSeq 2000	82078157	lymph node		GSM759509	female	Caucasian	86
	2x50 PE	ploy(A)-selected,	Illumina	80946260	ovary		GSM759511	female	African	47

		un-stranded	HiSeq 2000						American	
	2x50 PE	ploy(A)-selected, un-stranded	Illumina HiSeq 2000	82334076	prostate		GSM759513	male	Caucasian	73
	2x50 PE	ploy(A)-selected, un-stranded	Illumina HiSeq 2000	82111139	skeletal muscle		GSM759515	male	Caucasian	77
	2x50 PE	ploy(A)-selected, un-stranded	Illumina HiSeq 2000	81836199	testes		GSM759517	male	Caucasian	19
	2x50 PE	ploy(A)-selected, un-stranded	Illumina HiSeq 2000	81912887	thyroid		GSM759519	female	Caucasian	60
Mouse	2x50 PE	ploy(A)-selected, dUTP stranded-specific	Illumina HiSeq 2000	87264604	brain	1027 (21)	GSM1020640	male	DBA/2J	-
	2x50 PE	ploy(A)-selected, dUTP stranded-specific	Illumina HiSeq 2000	101816491	colon		GSM1020641	male	DBA/2J	-
	2x36 PE	ploy(A)-selected, dUTP stranded-specific	Illumina HiSeq 2000	35175982	heart		GSM1020642	male	DBA/2J	-
	2x50 PE	ploy(A)-selected, dUTP stranded-specific	Illumina HiSeq 2000	119274786	kidney		GSM1020643	male	DBA/2J	-
	2x50 PE	ploy(A)-selected, dUTP stranded-specific	Illumina HiSeq 2000	116292478	liver		GSM1020644	male	DBA/2J	-
	2x36 PE	ploy(A)-selected, dUTP stranded-specific	Illumina HiSeq 2000	34050626	lung		GSM1020645	male	DBA/2J	-
	2x50 PE	ploy(A)-selected, dUTP stranded-	Illumina HiSeq 2000	113111277	skeletal muscle		GSM1020646	male	DBA/2J	-

		specific								
	2x50 PE	ploy(A)-selected, dUTP stranded-specific	Illumina HiSeq 2000	114072257	spleen		GSM1020647	male	DBA/2J	-
	2x50 PE	ploy(A)-selected, dUTP stranded-specific	Illumina HiSeq 2000	109199938	testes		GSM1020648	male	DBA/2J	-
Rhesus monkey	2x80 PE	ploy(A)-selected, dUTP stranded-specific	Illumina HiSeq 2000	107669551	brain	2175 (8)	GSM1020702	male	-	-
	2x80 PE	ploy(A)-selected, dUTP stranded-specific	Illumina HiSeq 2000	104824449	colon		GSM1020703	male	-	-
	2x80 PE	ploy(A)-selected, dUTP stranded-specific	Illumina HiSeq 2000	109193093	heart		GSM1020704	male	-	-
	2x80 PE	ploy(A)-selected, dUTP stranded-specific	Illumina HiSeq 2000	108637672	kidney		GSM1020705	male	-	-
	2x80 PE	ploy(A)-selected, dUTP stranded-specific	Illumina HiSeq 2000	113094939	liver		GSM1020706	male	-	-
	2x80 PE	ploy(A)-selected, dUTP stranded-specific	Illumina HiSeq 2000	112732867	lung		GSM1020707	male	-	-
	2x80 PE	ploy(A)-selected, dUTP stranded-specific	Illumina HiSeq 2000	114963857	skeletal muscle		GSM1020708	male	-	-
	2x80 PE	ploy(A)-selected, dUTP stranded-	Illumina HiSeq 2000	97713278	spleen		GSM1020709	male	-	-

		specific								
	2x80 PE	ploy(A)-selected, dUTP stranded-specific	Illumina HiSeq 2000	115441819	testes		GSM102071 0	male	-	-

'-' denotes that the information is unknown.

Table S2. The proportion of strongly regulated AS GYNNGYs in mice increases linearly with the number of tissues within this range

Count of tissues	2	3	4	5	6	7	8	9
Average Percentage (%)	2.225397	0.01908196	2.047416	2.231317	2.393152	2.517935	02.604378	2.710027

Table S3. Spearman correlations between UMS of each AS GYNNGY and expression level of the gene containing the site.

(A) Human

	CDS			UTR		
**Tissue**	**P**	**Rho**	**Site count**	**P**	**Rho**	**Site count**
**Adipose**	6.52E-09	-0.6196	72	0.074921	-0.26516	46
**Adrenal**	7.90E-05	-0.37723	104	0.52955	-0.10672	37
**Brain**	0.000147	-0.36711	102	0.841107	-0.03893	29
**Breast**	1.76E-04	-0.40506	81	0.922337	0.020769	25
**Colon**	5.89E-06	-0.51197	70	0.373115	-0.18154	26
**Heart**	1.11E-06	-0.5222	77	0.712322	-0.06097	39
**Kidney**	3.88E-06	-0.5018	76	0.100095	-0.31141	29
**Leukocyte**	2.01E-05	-0.54533	54	0.107889	-0.29948	30
**Liver**	2.18E-09	-0.65639	66	0.023159	-0.53767	18
**Lung**	1.23E-05	-0.55237	55	0.131874	-0.29181	28
**Lymph node**	0.001152	-0.39728	64	0.108876	-0.35178	22
**Ovary**	6.60E-08	-0.44307	136	0.423736	-0.11449	51
**Prostate**	1.08E-08	-0.52037	106	0.04023	-0.37044	31
**Skeletal muscle**	2.43E-04	-0.50135	49	0.868247	-0.03421	26
**Testis**	1.07E-05	-0.36894	135	0.193791	-0.18497	51
**Thyroid**	4.87E-11	-0.53098	133	0.141779	-0.19885	56

(B) Mouse

	CDS			UTR		
**Tissue**	**P**	**Rho**	**Site count**	**P**	**Rho**	**Site count**
**Brain**	5.07E-12	-0.51237	159	0.215562	-0.20017	40
**Colon**	4.59E-21	-0.62307	183	0.026103	-0.39479	32
**Heart**	0.905038	0.038665	12	0.563889	0.314286	6
**Kidney**	1.30E-15	-0.53504	192	0.045593	-0.29956	45
**Liver**	6.74E-22	-0.67594	154	0.022359	-0.43006	28
**Lung**	0.02288	-0.50583	20	0.278965	0.381818	10
**Skeletal muscle**	4.63E-15	-0.62011	129	0.964603	-0.00957	24
**Spleen**	6.20E-12	-0.44286	219	0.020531	-0.37948	37
**Testis**	1.03E-28	-0.57378	312	0.906887	-0.01468	66

Table S4. Counts of GYNNGYs after eliminating strongly regulated ones

	Human		Mouse	
	CDS	UTR	CDS	UTR
**AS GYNNGY**	546	132	830	176
**All GYNNGY**	107146	14934	109891	14154
**AS proportion**	0.51%	0.88%	0.76%	1.24%
**P value**[a]	1.146e-8		4.032e-13	

a: P value of Fisher's exact test whether UTR has significantly higher proportion of AS GYNNGYs than CDS.

Table S5. Evaluation of conservation of alternative GYNNGYs between human and mouse using sequence identity.

		Strong	Medium	Weak	Very weak	Unregulated
**Human**	**Conserved**	30	24	11	7	62
	**Total**	73	64	38	41	279
	**Proportion of conserved**	41.10%	37.50%	28.95%	17.07%	22.22%
**Mouse**	**Conserved**	9	21	17	12	92
	**Total**	25	52	50	59	456
	**Proportion of conserved**	32%	26.92%	18.00%	15.25%	10.31%

The same as Table 3, except that a human AS GYNNGY was considered conserved in mice if the orthologous region had the same sequence regardless of its splicing status. The strongly regulated AS of GYNNGYs is more conserved than the combination of the other groups (Chi-square test, P = 0.005465 and P = 0.1492 for humans and mice, respectively).

Table S6. Sequence motifs in the downstream 50nt intronic regions of AS GYNNGYs. In total, 118 strongly regulated, 159 less regulated, and 342 unregulated AS GYNNGYs are used. Two E-value cutoffs were used in motif scan by fimo, E-value <= 1e-4 and <=1e-3. For the latter, the values are shown in parentheses.

Motif index	Motif logo	Number of regions with the given motif			P values (Fisher's exact test)	
		Strongly regulated	Less regulated	Unregulated	Strongly vs. Less	Strongly vs. unregulated
1	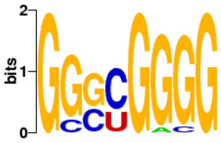	19 (33)	14 (44)	47 (111)	0.09024 (1)	0.5438 (0.4206)
2	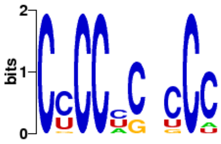	14 (18)	8 (24)	20 (63)	0.04428 (1)	0.0405 (0.4855)
3	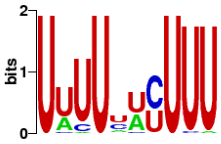	**12 (25)**	**2 (14)**	**12 (37)**	**0.00125 (0.004792)**	**0.008037 (0.007326)**
4	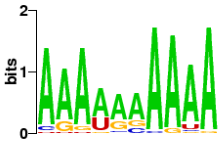	4 (4)	0 (6)	1 (4)	0.03197 (1)	0.01667 (0.2125)
5	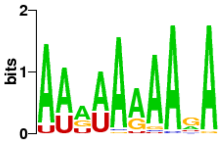	4 (4)	1 (6)	1 (13)	0.1671 (1)	0.01667 (1)
6	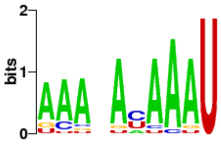	2 (3)	0 (3)	1 (9)	0.1806 (0.7021)	0.163 (1)
7	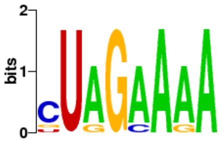	2 (3)	0 (0)	0 (4)	0.1806 (0.07617)	0.06539 (0.3798)
8	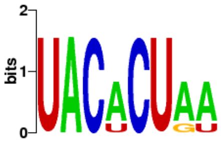	2 (2)	0 (2)	0 (1)	0.1806 (1)	0.06539 (0.163)

Table S7. Comparison of UMS between AS GYNNGYs and AS NAGNAGs

Site group	Mean UMS of GYNNGY*	Mean UMS of NAGNAG*	Wilcoxon rank sum test, P values
Strong	0.1758 (118)	0.2384 (741)	3.89e-07
Medium	0.0294 (73)	0.0451 (222)	0.00458
Weak	0.0089 (43)	0.0137 (72)	0.0110
Very Weak	0.0028 (43)	0.0038 (64)	0.236
Unregulated	0.0260 (344)	0.0904 (788)	6.07e-21

*: In parentheses are counts of studied sites in each case.

Table S8. Proportions of AS GYNNGYs showing mean UMS ≥0.05 in different groups

(A) Human

	Strong	Medium	Weak	Very weak	Unregulated
Mean UMS >=0.05	94	13	0	0	43
Total	118	73	43	43	344
Proportion	79.66%	17.81%	0	0	12.50%

(B) Mouse

	Strong	Medium	Weak	Very weak	Unregulated
Mean UMS >=0.05	21	4	1	0	80
Total	21	16	6	7	687
Proportion	100%	25%	16.67%	0	11.64%

In each cell, counts of GYNNGYs or proportions are given for each category

Table S9. Reanalysis of human NAGNAGs

	Analysis of all AS events		Analysis after excluding AS events that do not have all isoforms' PSI ≥ 5% in at least one tissue
Strongly regulated	741		744
Medium	222		228
Weak	72		75
Very weak	64		1
Unregulated	865		476
Testable	1887		1524
Proportion of strongly regulated	39.27%		48.82%

Note. The classification of the four groups is the same as for GYNNGYs. The testable ones are those with reads >= 10 in at least two tissues. In total, 10211 NAGNAG splice sites were found in the genome and 2438 (19.27%) are alternatively spliced.

Table S10. The gain and loss of alternatively spliced GYNNGYs and NAGNAGs
(A) Analysis using all AS GYNNGY and AS NAGNAG events detected in either human or mouse.

Human	Rhesus	Mouse	5' GYNNGY	3' NAGNAG
-	-	+	571	1171
+	-	-	212	479
+	-	+	16	127
-	+	-	1570	1019
-	+	+	108	222
+	+	-	252	677
+	+	+	89	691

Note. "+" and "-" denote the presence and absence of an AS event in a certain organism for an orthologous region of GYNNGY or NAGNAG. Human specific alternative GYNNGY ratio is significantly higher than that of alternative NAGNAG (37.26% to 24.27%, Chi-square test P = 1.166e-09). Similarly, rhesus specific alternative GYNNGY ratio is also higher than alternative NAGNAG (77.76% to 39.06%, Chi-square test P < 2.2e-16). Meanwhile, the loss ratios are inconsistent: in human, higher GYNNGY loss ratio than NAGNAG is observed (18.98% to 11.25%, Chi-square P = 4.133e-05); and in rhesus, the result is different (0.79% to 4.87%, Chi-square < 2.2e-16).

(B) Similar as (A), but analysis is restricted to genes with both GYNNGY and NAGNAG splice sites.

Human	Rhesus	Mouse	5' GYNNGY	3' NAGNAG
-	-	+	202	320
+	-	-	64	105
+	-	+	3	33
-	+	-	569	267
-	+	+	38	59
+	+	-	87	153
+	+	+	22	184

Human specific alternative GYNNGY ratio is significantly higher than that of alternative NAGNAG (36.36% to 22.11%, Chi-square test P = 0.0003373). Similarly, rhesus specific alternative GYNNGY ratio is also higher than alternative NAGNAG (79.47% to 40.27%, Chi-square test P < 2.2e-16). Meanwhile, the loss ratios are inconsistent: in human, higher GYNNGY loss ratio than NAGNAG is observed (21.59% to 12.42%, Chi-square P = 0.01889); and in rhesus, the result is different (0.42% to 4.98%, Fisher's exact test < 6.128e-08).

Table S11. Estimates of effects of gender and age on splicing regulation.

Factors	Study	Number of tissue types used in the study (**A**)	Number of affected splicing events (**B**)	Percentage of alternative 5’ splice sites of all AS events* (**C**)	Percentage of Δ4# events of all AS events at 5’ splice sites* (**D**)	Estimated number of Δ4 events due to the considered factor (**E**)
Gender	(44)	12	395	6.64%	30%	10.5
Age	(45)	2	1484	6.64%	30%	236.5

*: these two numbers were estimated based on the results in the studies (5) and (19), respectively. #: Δ4 indicates the cases where the two tandem splice sites in an alternative 5’ splicing event are 4nt apart. The estimate of Δ4 alternative 5’ splice sites in 16 human tissues, E, is calculated as E = B x C x D x 16/A. Details of the estimating procedure is as follows.

To estimate the effect of gender on splicing variation, we used the data from a study of 12 human brain regions of 137 individuals (44). The study identified 395 genes with sex-biased splicing and we treated this number as the number of splicing events by assuming each gene had one sex-biased event. Then according to Wang, et al. (5), 6.64% of alternative splicing events result from alternative 5’ splice sites, and according to Bortfeldt, et al. (19), ~30% of alternative 5’ splice sites are Δ4 type. Therefore we could estimate the number of Δ4 alternative 5’ splice sites using the above formula, assuming that each tissue type contributes the same number of different splicing events.

In a similar way, we estimated the effect of age on splicing variation. The data was from a brain development study (45), which studied individuals in the age range of 0 to 98 and identified 1484 alternative spliced regions that varied along brain development. In this study, two brain regions were studied.

In general, these estimates are very rough and may be over- or under- estimate depending on the studied samples and the assumptions.

Table S12. Translational efficiency of the two splicing isoforms of the gene *NDUFS5* from HEK293 cells

Biochemical condition	Relative translational efficiency of NM_004552 (TIS-seq reads; RNA-seq reads)	Relative translational efficiency of NM_001184979 (TIS-seq reads; RNA-seq reads)
LTM	10.7 (107; 10)	2.4 (57; 24)
CHX	2.5 (25; 10)	0.13 (3; 24)

TIS-seq measures ribosome density at translation initiation site (ribosomes blocked by chemical LTM or CHX). Hence TIS-seq read counts are proportional to translation activity. RNA-seq measures RNA abundance, Therefore, the ratio of these two measures (TIS-seq reads/RNA-seq reads) gives the relative translational efficiency (see Supplementary methods for details).

Supplementary Datasets (see separate files)

Dataset S1. Human detected alternative GYNNGY information.

Dataset S2. Mouse detected alternative GYNNGY information.

Supplementary Figures

Figure S1. Comparison of maximum UMS between CDS and UTR regions in humans (A) and mice (B). For each AS GYNNGY 5' splice site, tissues with reads ≥ 6 were considered, and the maximum UMS among tissues was calculated. The maximum UMS is significantly lower in CDS than in UTR (Wilcoxon rank sum test, P = 1.5e-11 and 6.3e-16 for humans and mice, respectively).

Figure S2. In mice, the UMS of GYNNGY splice sites in CDS is positively correlated with dN/dS ratio (Panel A), and negatively correlated with mean gene abundance (Panel C) (Spearman's Rho = 0.12, P = 0.001, and Rho = -0.49, P < 2.2e-16, respectively). The UMS in UTR has similar but less significant relationships with these variables (B and D, Rho = 0.11, P = 0.19 and Rho = -0.20, P = 0.0122).

Figure S3. The counts of strongly regulated AS events showing maximum in each tissue. The horizontal line indicates the expected count for each tissue assuming uniformity. Heart and ovary tissues have significantly fewer counts than expected (Two-tailed Binomial test, P < 0.01).

Figure S4. The proportions of strongly regulated sites increase with the number of examined tissues in (A) humans and (B) mice. 95% confidence intervals are shown, estimated under Student's t-distribution. The red line in (A) is the fitted curve (see Materials and methods).

Figure S5. The magnitudes of absolute splice score difference between the two tandem splice sites in each GYNNGY is negatively correlated with the degree of regulation (given by the ΔUMS) (For sites with FDR ≤ 0.01, spearman's Rho = -0.21, P = 0.0006).

(continue to next page)

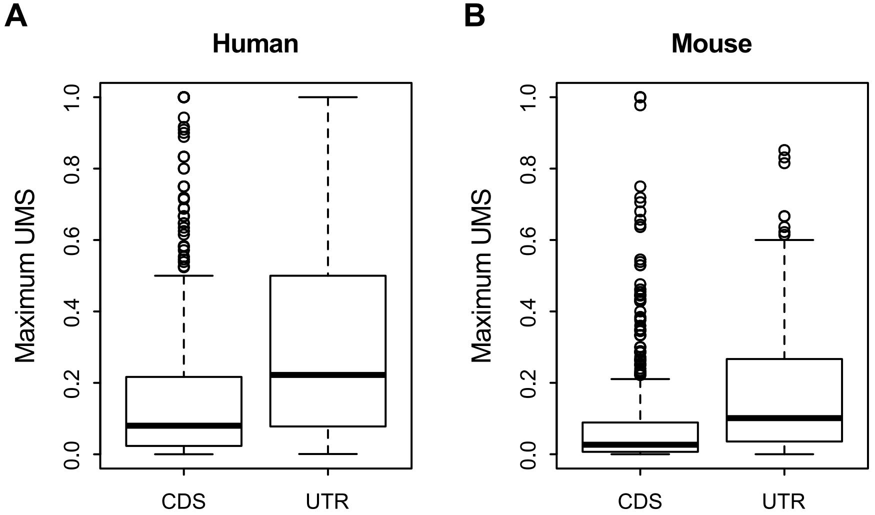
A
Human
Maximum UMS
1.0
0.8
0.6
0.4
0.2
0.0
CDS
UTR
B
Mouse
Maximum UMS
1.0
0.8
0.6
0.4
0.2
0.0
CDS
UTR

Figure S1

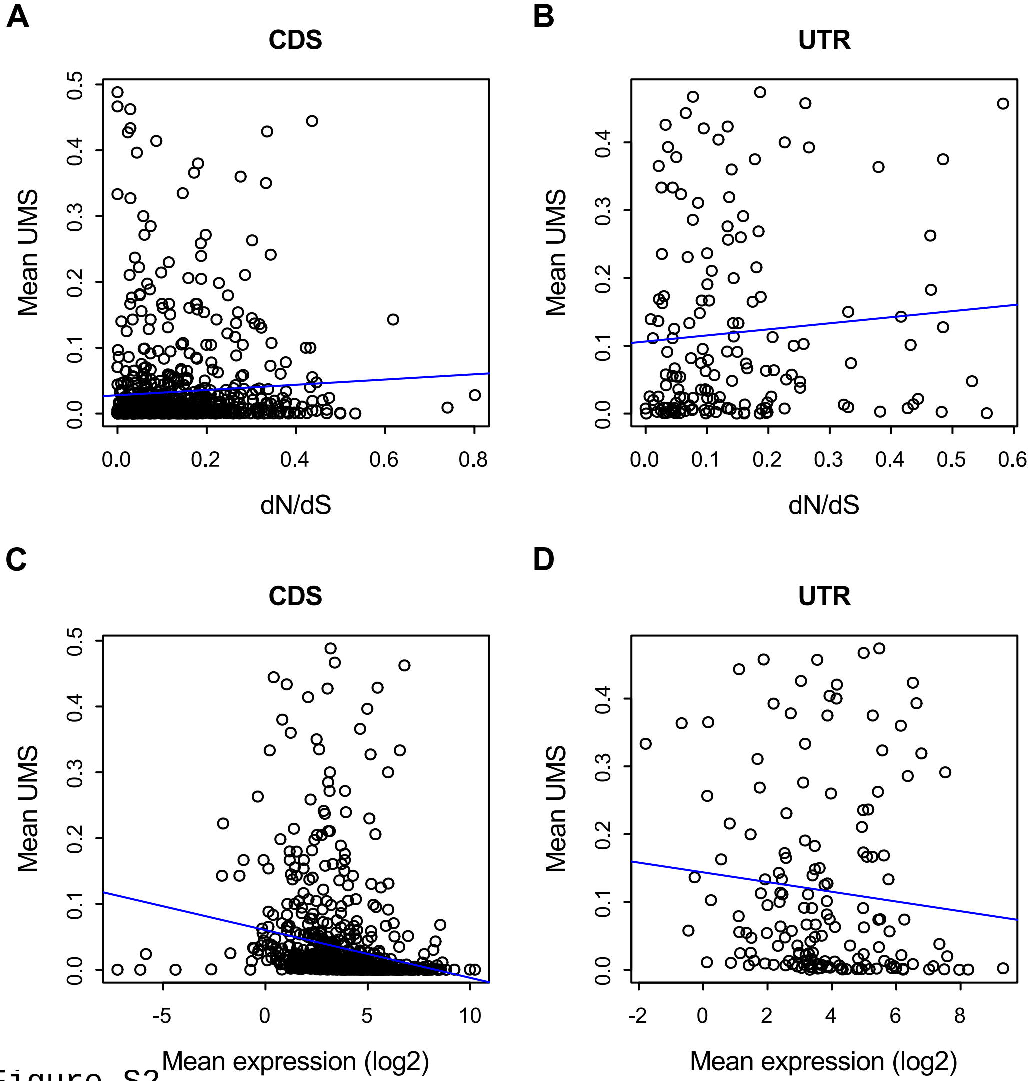
A
CDS
Mean UMS
dN/dS
B
UTR
Mean UMS
dN/dS
C
CDS
Mean UMS
Mean expression (log2)
D
UTR
Mean UMS
Mean expression (log2)

Figure S2

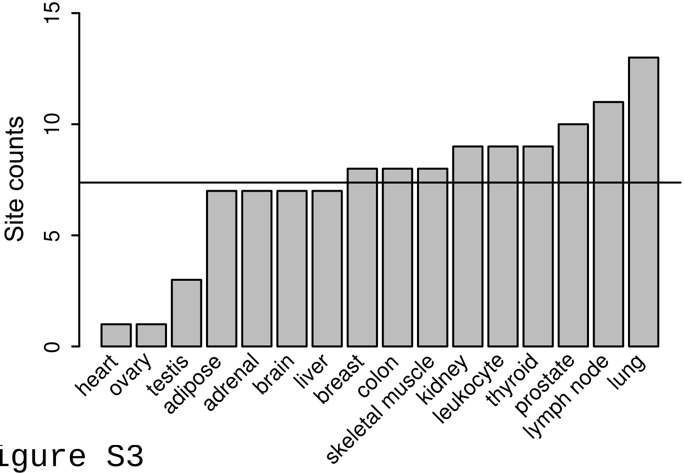

Site counts
0
5
10
15
heart
ovary
testis
adipose
adrenal
brain
liver
breast
colon
skeletal muscle
kidney
leukocyte
thyroid
prostate
lymph node
lung

Figure S3

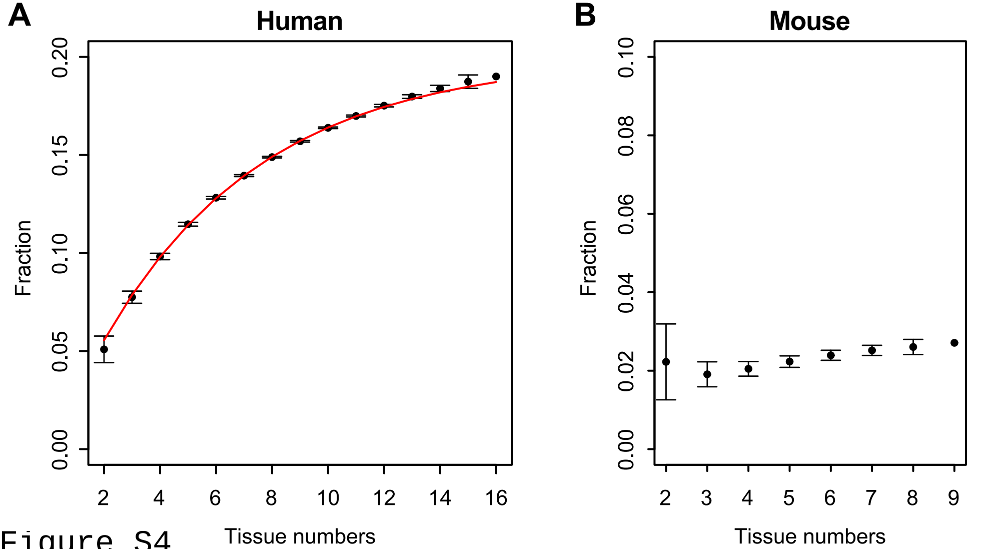
A
Human
0.00
0.05
0.10
0.15
0.20
Fraction
2
4
6
8
10
12
14
16
Tissue numbers
B
Mouse
0.00
0.02
0.04
0.06
0.08
0.10
Fraction
2
3
4
5
6
7
8
9
Tissue numbers

Figure S4

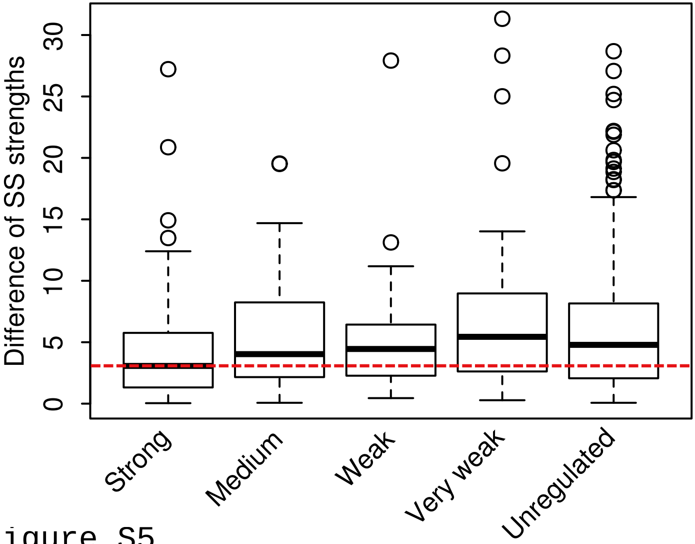

Difference of SS strengths
0
5
10
15
20
25
30
Strong
Medium
Weak
Very weak
Unregulated

Figure S5
